# HOXA1 Contributes to Bronchial Epithelial Cell Cycle Progression by Regulating p21/CDKN1A

**DOI:** 10.3390/ijms26052332

**Published:** 2025-03-05

**Authors:** Elizabeth McCluskey, Sathesh Kanna Velli, Rafal Kaminski, Tyler Markward, Hannah Leming, Daohai Yu, Umadevi Sajjan

**Affiliations:** 1 Center for Inflammation and Lung Research, Lewis-Katz Medical School, Temple University, Philadelphia, PA 19140, USAsathesh.kanna@temple.edu (S.K.V.); tyler.markward@temple.edu (T.M.); hannah.leming@temple.edu (H.L.); 2 Center for Neurovirology and Gene Editing, Lewis-Katz Medical School, Temple University, Philadelphia, PA 19140, USA; rafal.kaminski@temple.edu; 3 Center for Biostatistics and Epidemiology, Lewis-Katz Medical School, Temple University, Philadelphia, PA 19140, USA; 4 Department of Thoracic Medicine and Surgery, Temple University Health System, Philadelphia, PA 19140, USA

**Keywords:** homeobox A1, cell proliferation, cell migration, airway epithelial repair, COPD

## Abstract

Airway basal cells proliferate and regenerate airway epithelium after injury. The first step during airway epithelial repair is airway basal cell proliferation to close the wound. Previously, we demonstrated that *homeobox (HOX) A1* expression is reduced in airway stem cells isolated from chronic obstructive pulmonary disease. *HOXA1* is a developmental gene and plays a role in hematopoietic stem cell proliferation and differentiation, but its contribution to airway epithelial cell migration and proliferation is not known. In this study, we generated a HOXA1 knockout bronchial epithelial cell line using CRISPR/CAS9 technology followed by clonal expansion to investigate the role of HOXA1 in airway epithelial cell proliferation and migration. Compared to WT, HOXA1 knockout bronchial epithelial cells generated smaller spheroids than WT type cells, indicating a defect in cell proliferation. In the scratch assay, HOXA1 knockout cells showed substantial delay in migrating to the wounded area. By single-cell RNA sequencing and the clustering of cells based on HOXA1 expression, we identified a downregulation of genes involved in cell cycle progression. A cell cycle analysis by flow cytometry indicated partial cell cycle arrest at the G0/G1 phase in HOXA1 knockout cells. This was associated with a reduced expression of Cyclin E1 and an increased expression of the cyclin-dependent kinase inhibitor p21/CDKN1A. These results indicate that *HOXA1* may contribute to cell proliferation by regulating cell cycle progression via p21/CDKN1A in airway epithelial cells.

## 1. Introduction

The airway epithelium is the first line of defense and protects the lungs from environmental insults. The basal cells present in the airway epithelium are tissue-specific stem cells and repair the airway epithelium quickly following injury to protect the lungs [1,2]. The migration and proliferation of basal cells are the first step during the repair of the airway epithelium, and the mechanisms involved are not completely understood, particularly under a chronic disease state such as chronic obstructive pulmonary disease (COPD).

Previously, we and others have demonstrated that airway basal cells from patients with COPD regenerate the airway epithelium with an abnormal phenotype [3,4,5,6,7]. Airway epithelial repair transcription factors, such as ETS homologous factor, NOTCH, Wnt-β, grainyhead-like2, and YAP1, have been demonstrated to contribute to basal cell proliferation and airway epithelial repair [8,9,10,11,12,13,14,15], but they do not appear to play a role in the abnormal repair of the airway epithelium in COPD.

Previously, we demonstrated that compared to normal, COPD airway basal cells show a downregulation in some developmental genes including homeobox *(HOX)A1* [5,16]. *HOX* genes are transcription factors and primarily expressed during embryonic development. Interestingly, *HOX* gene expression is also observed in adult tissues and is thought to be required for the continuous reassessing of positional values to maintain correct cell identities and promote the repair/regeneration of tissue (Morgan, 2006 #7129) [17]. This is especially true for cells that undergo frequent renewal, such as adult hematopoietic stem cells, fibroblasts, skeletal and skin stem cells, endometrial cells of the uterus, and endothelial cells during vascular remodeling [17,18,19,20,21,22]. HOXA1 has been shown to contribute to adult hematopoietic stem cell proliferation and differentiation [23,24]. The expression of *HOX* genes, including *HOXA1*, was observed in adult human lungs [25], indicating they may play a role in adult lung repair. Since *HOXA1* is expressed in airway basal cells [5], we hypothesized that *HOXA1* may contribute to the early phase of repair that is the migration and proliferation of these cells.

We generated a *HOXA1* knockout bronchial epithelial cell line to demonstrate that HOXA1 contributes to cell proliferation and migration. We also demonstrated that HOXA1 plays a role in promoting cell cycle progression.

## 2. Results

### 2.1. Generation of HOXA1 Knockout 16HBE14o- Cells

Bronchial epithelial cell line 16HBE14o- cells were derived from the bronchi of healthy non-smokers, retaining the epithelial cell phenotype during passaging. CRISPR/CAS9 technology was used to generate the *HOXA1* knockout (K/O) 16HBE14o- cell line. Two sgRNAs targeting two areas in Exon-1 of the *HOXA1* gene were used to excise 201 bp of the gene (Supplementary Figure S1). This method generated a mixture of cells showing both the wild type and a truncated *HOXA1* gene as determined by the PCR analysis on genomic DNA and by sequencing the PCR products.

### 2.2. Single-Cell RNAseq Analysis of HOXA1 Knockdown Cells

**Figure 1:**
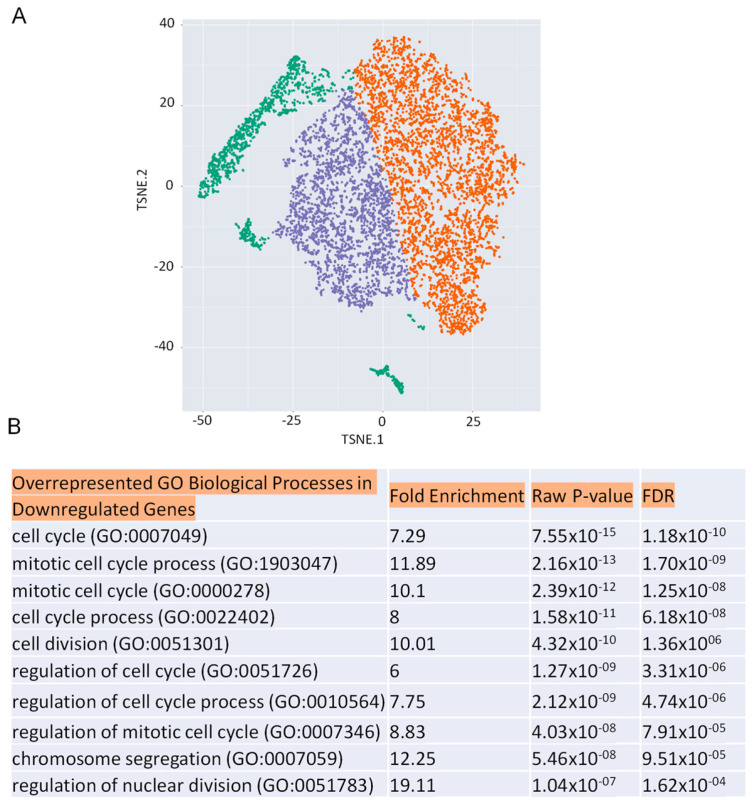
Clustering of cells based on *HOXA1* expression. (**A**). The cells were clustered based on *HOXA1* expression on the Rosalind platform using Seurat. The differentially regulated genes were identified by comparing the gene expression between low (purple dots) and high (orange dots) *HOXA1* expressing cells. The green dots represent cells with <200 reads per cell and were excluded from the analysis. (**B**). The gene ontology of downregulated genes in *HOXA1* identified biological processes involved in cell cycle progression.

To identify the potential pathways regulated by *HOXA1* in airway epithelial cells, we conducted a single-cell RNAseq analysis using a mixture of WT and *HOXA1* knockdown cells. The UMAP analysis grouped the cells into several clusters based on gene expression. Interestingly, based on *HOXA1* gene expression, the cells clustered into three groups: no, low, or high *HOXA1* expression (Figure 1A). The quality of the reads for cells expressing no *HOXA1* was very poor (green dots); therefore, we compared gene expressions between low (blue dots) and (orange dots) high *HOXA1*-expressing cells to identify differentially regulated genes. Only 94 transcripts were differentially expressed with a *p*-value of ≤0.05 and a fold change > 1.3 (Supplementary Table S1). Out of 94 transcripts, 53 were downregulated, and the gene ontology analysis of these transcripts indicated the enrichment of biological processes related to cell cycle regulation, with FDR ranging between 1.18 × 10^−10^ and 1.62 × 10^−4^ (Figure 1B), suggesting a role for *HOXA1* in cell cycle progression and proliferation. *HOXA1* K/O cells also showed downregulation in actin binding protein anillin and CDC42 effector binding protein 1, several Kinesin family members which are involved in the migration of cells.

### 2.3. Clonal Expansion of HOXA1 Knockdown Cells

**Figure 2:**
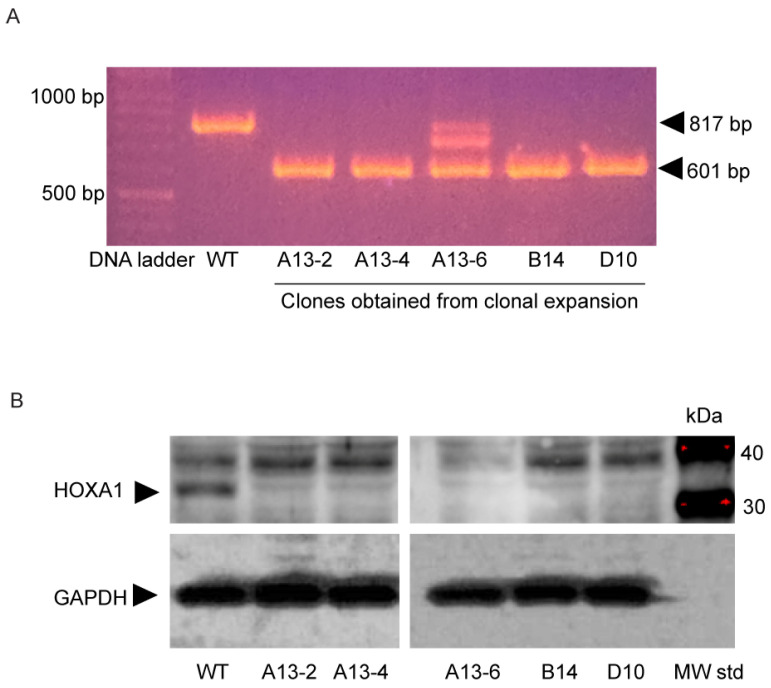
Clonal expansion of CRISPR mutants to isolate *HOXA1* K/O clone. Mixture of cells obtained after sgRNA transfection was seeded at one cell per well in 96 well plates. (**A**) Five clones showing only the truncated HOXA1 band were selected and passaged twice and reanalyzed by PCR. (**B**) Total protein was isolated from all five clones and subjected to Western blot analysis. All the clones showed no HOXA1 protein, indicating the efficient knockout of HOXA1 at the protein level. The red dots in MW std lane represent over saturation of molecular weight standards.

To determine the role of HOXA1 in proliferation, we isolated pure *HOXA1* knockout (K/O) clones by clonal expansion, since a mixture of clones generated from CRISPR/CAS9 technology gave inconsistent results. By clonal expansion, we obtained several clones with the truncated *HOXA1* gene (Figure 2A), and one clone showed two additional bands. By Western blot analysis, we found that all the clones except for A13-6 showed a band at 40 kDa, which is likely a non-specific band. In contrast, the HOXA1 band at 37 kDa was only observed in WT, but not in *HOXA1* K/O clones (Figure 2B). A13-2 was selected for subsequent studies because this clone showed the consistent deletion of HOXA1 at the protein level through several passages. In the rest of the experiments, the A13-2 clone was referred to as HOXA1 K/O cells.

### 2.4. HOXA1 K/O Cells Show Delay in Wound Closure

**Figure 3:**
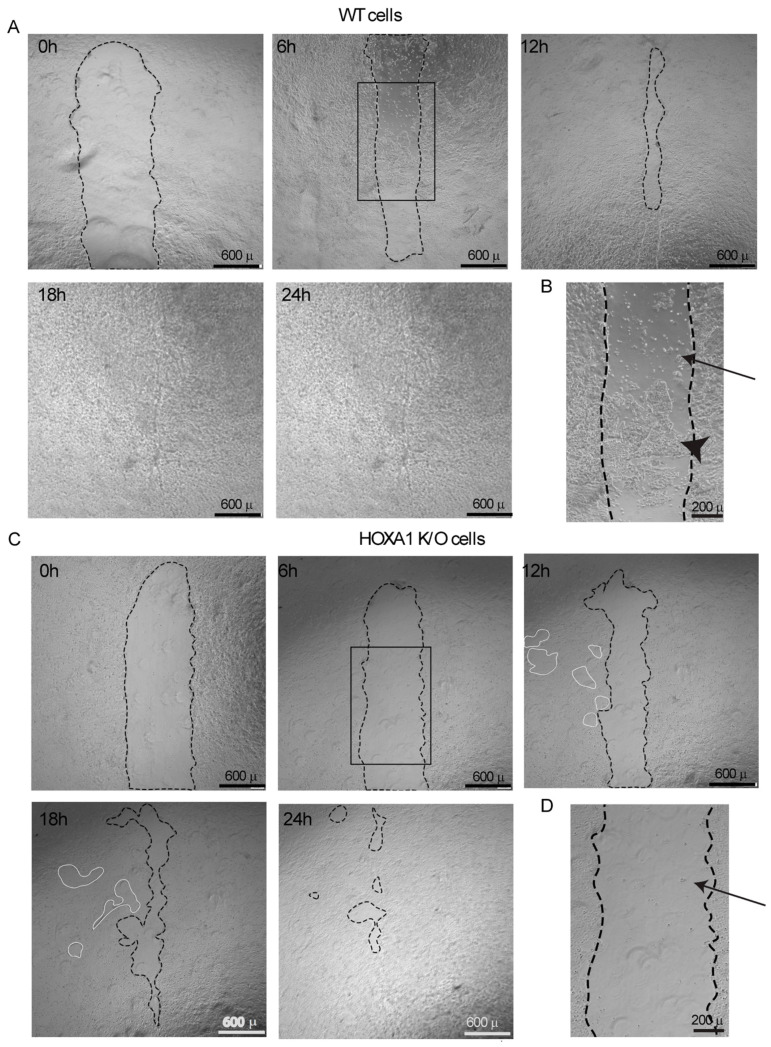
HOXA1 K/O cells show delayed wound healing compared to WT cells. Both WT and HOXA1 K/O cells were grown to 100% confluency in 24-well plates. Mechanical wounds of approximately 1 mm × 6 mm were created, cells were washed with PBS, and the medium was replaced with a fresh complete medium. The cultures were imaged under a phase contrast microscope at 0, 6, 12, 18, and 24 h. (**A**,**C**) The cultures were imaged under a phase contrast microscope at 0, 6, 12, 18, and 24 h. The areas marked in black dotted lines in images represent wounds in the cell cultures. The areas marked with white dotted lines in (**C**) represent spontaneous wound formation adjacent to the original wound. (**B**,**D**) The images are the magnified areas marked in rectangle in (**A**) and (**B**), respectively. Arrows and arrowheads respectively represent single cells and large colonies of cells. Images are representative of 3 independent experiments performed in triplicate.

In cancer cells, HOXA1 plays a critical role in cell migration and proliferation [26,27]. Cell migration and proliferation play an important role in the repair of the airway epithelium. To examine whether HOXA1 contributes to cell migration and proliferation of airway epithelial cells, we conducted a wound closure assay. Both WT and HOXA1 K/O cells were seeded at a high density to obtain 100% confluent monolayers, created similar size wounds, and monitored wound closure by time-lapse microscopy. In WT cell cultures, the wounds completely closed by 18 h (Figure 3A). But in HOXA1 K/O cell cultures, large, wounded areas were observed up to 18 h post-injury. At 24 h post-injury, HOXA1 K/O cells showed small, wounded areas (Figure 3C). The quantification of wound size indicated a significant difference in the rate of wound closure between WT and HOXA1 K/O cells at all time points measured (Supplementary Figure S2). Interestingly, we found that WT cells showed an abundant number of large clusters of cells in the wounded site as early as 6 h after the injury (Figure 3B). In contrast, HOXA1 K/O cells showed a few individual cells in the wounded area at 6 h post-injury (Figure 3D). Another important feature that we observed in HOXA1 K/O cells was the appearance of new small wounds adjacent to the original wound at 12 and 18 h post-injury, which eventually closed by 24 h (Figure 3B, white dotted lines). These results indicate that HOXA1 K/O cells may have defects in both migration and proliferation after injury, and this may contribute to delayed wound closure.

### 2.5. HOXA1 Plays a Role in Cell Proliferation

**Figure 4:**
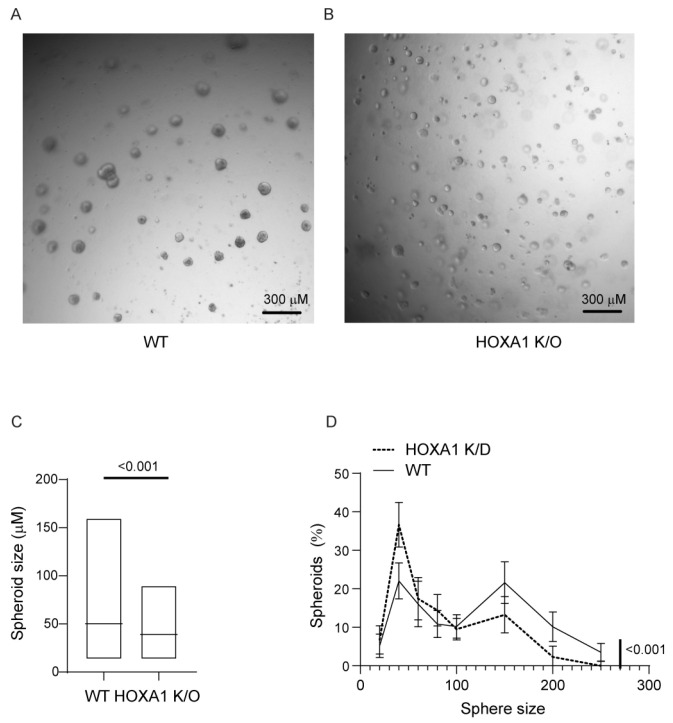
HOXA1 K/O cells show defects in proliferation. WT control and HOXA1 K/O cells at passage 3 or 5 were suspended in a complete medium and mixed 1:1 with 100% Matrigel^®^ with reduced growth factors and 100 µL containing 1000 cells in 6 mm trans wells. The medium was changed every other day for 7 days and imaged under a phase contrast microscope. Two to three random fields per culture were used to determine the number and size of the spheres. (**A**,**B**) Representative image of WT and HOXA1 K/O cells, respectively. (**C**) The data represent a range with the median from three independent experiments performed in triplicate (*p* ≤ 0.001, Mann–Whitney test). (**D**) The distribution of spheres based on the size and the statistical difference between WT and HOXA1 K/O cells were determined by the Kolmogorov–Smirnov test for variable diameter. Data represent mean with SD.

To assess the role of HOXA1 in cell proliferation, we conducted a sphere forming assay. In this assay, the cells were seeded at a low density in Matrigel with reduced growth factors, and therefore, each spheroid was generated from a single cell. The number and size of the colonies were measured after 7 days of seeding. The number of spheroids indicated viable cells capable of generating spheroids, and the size of the spheroids indicated the capacity of the cell to proliferate. Compared to WT, HOXA1 K/O cells showed a similar number of spheres, but the size of the spheres was smaller (Figure 4A,B). When the overall size of the spheres was assessed, HOXA1 K/O cells showed significantly smaller sized spheres than WT (Figure 4C). The Kolmogorov–Smirnov test for variable diameter indicated a significant difference between WT and HOXA1 K/O in the distribution of different sized spheres (Figure 4D).

A smaller spheroid size indicates attenuation in cell proliferation. To assess whether the attenuated cell proliferation in HOXA1 K/O cell cultures was due to increased cell death, we quantified the apoptotic and necrotic cells by flow cytometry. There was no significant difference between WT and HOXA1 K/O in apoptotic (annexin +ve), late apoptotic (annexin and propidium iodide +ve), or necrotic (propidium iodide +ve) cells (Supplementary Figure S3A–D). These results indicate that the reduction in cell proliferation is not due to increased cell death in HOXA1 K/O cells.

### 2.6. HOXA1 Contributes to Cell Cycle Progression

**Figure 5:**
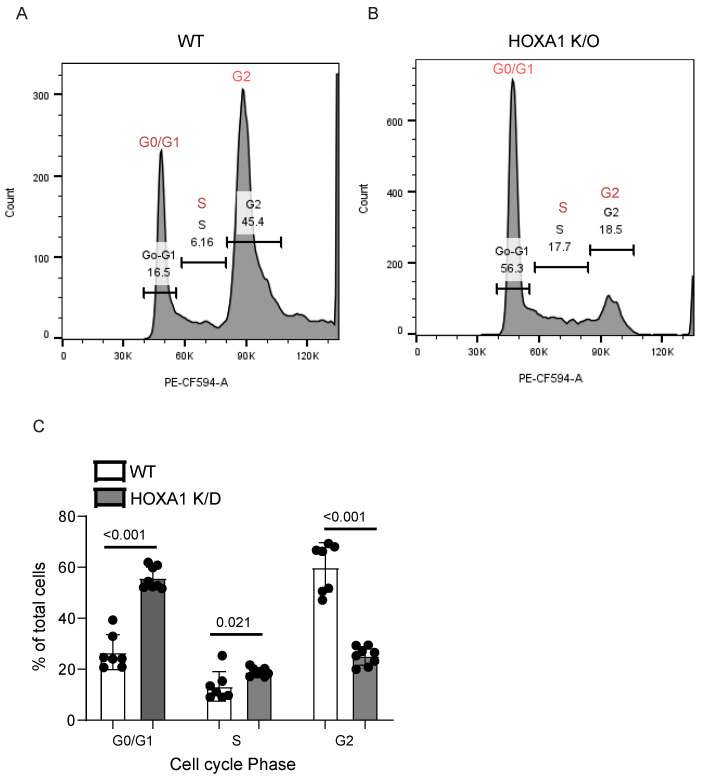
HOXA1 K/O cells show cell cycle arrest at the G0/G1 phase. An equal number of WT and HOXA1 K/O cells were seeded in 6 mm dishes and cultured for 48 h with one medium change at 24 h post-seeding. The cells were harvested, fixed in cold methanol, treated with RNase A, stained with propidium iodide, and analyzed with flow cytometry. The data were analyzed by FlowJo V10. (**A**,**B**) Representative histograms of WT and HOXA1 K/O cells showing cells at a different phase of cell cycle. (**C**) Percentage of total cells at G0/G1, S, and G2 cells in WT and HOXA1 K/O cells. Data represent range with a median calculated from three independent experiments with 2–3 replicates. Statistical significance between WT and HOXA1 K/O cells in each phase was determined by Mann–Whitney test.

A single-cell RNAseq analysis indicated the downregulation of genes associated with cell cycle progression, which could affect the cell proliferation and wound closure. Therefore, we assessed the cell cycle progression in WT and HOXA1 K/O cells by a propidium iodide staining assay (Figure 5A,B). The gating strategy is shown in Supplementary Figure S4. Compared to WT, HOXA1 K/O cell cultures showed a significantly higher percentage of cells in G0/G1 and a lower percentage of cells in the G2 phase (Figure 5C). These results indicated that HOXA1 may be required for the progression of cell cycles through the G1/G0 phase. Interestingly, HOXA1 K/O cell cultures also showed higher-percentage cells in the S phase than WT ones, indicating HOXA1 K/O cells may not have defects in DNA replication, which occurs during the S phase.

**Figure 6:**
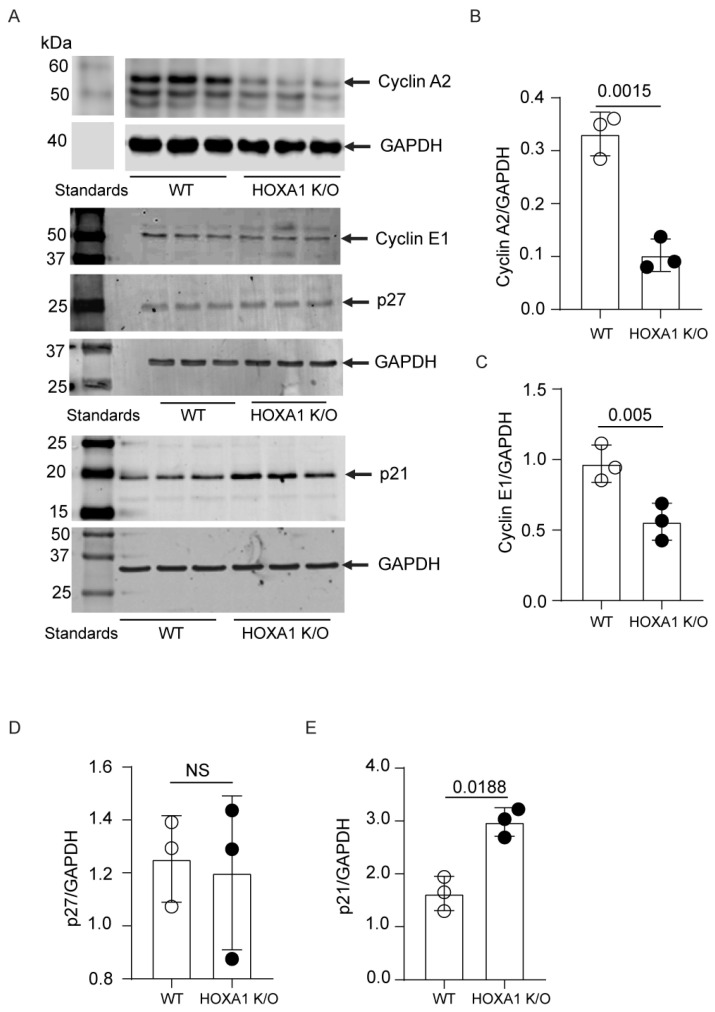
Expressions of Cyclin E1 and A2 were decreased in HOXA1 K/O cells. (**A**) WT and HOXA1 cells were grown to 50 to 60% confluency, washed with cold PBS, and lysed in RIPA buffer. The lysates were centrifuged, and the total protein was quantified in a supernatant. An equal amount of total protein was subjected to Western blot analysis using antibodies to Cyclin E1, Cyclin A2, P27, or P21. The blots were stripped and reprobed with antibodies to GAPDH. (**B**–**E**) The band densities were quantified by ImageJ (version 1.54) and expressed as fold change over GAPDH. NS, nonsignificant (*p* ≥ 0.05).

To investigate the possible mechanisms of cell cycle arrest, we determined the levels of Cyclin E1, which plays a role in transitioning cells from the G1 to S phase, and Cyclin A2, which promotes the transitioning of cells from the S to G2 phase. We also determined the levels of p21/CDKN1A and p27/CDKN1B proteins, which negatively regulate cell cycle progression from the G0/G1 to S phase and the S to G2 phase. Compared to WT ones, HOXA1 K/O cells showed a significant reduction in the expression of both Cyclin E1 and Cyclin A2 by Western blot analysis (Figure 6A–C). While p21 expression was significantly higher in HOXA1 K/O cells than in WT cells, there was no difference in the expression of p27 between WT and HOXA1 K/O cells (Figure 6A,D,E). Together, these results suggest that HOXA1 contributes to cell proliferation by regulating cell cycle progression, probably via the negative regulation of p21/CDKN1A.

## 3. Discussion

Cell proliferation is the first step during the repair of the airway epithelium after injury. Here, we demonstrate that HOXA1 contributes to cell proliferation by regulating the cell cycle progression in bronchial epithelial cells. Mechanistically, HOXA1 is required for the transition of cells from the G1 to S phase of the cell cycle, and the deletion of HOXA1 attenuates this transition by increasing the expression of p21/CDKN1A. As far as we know, this is the first report to demonstrate the role of HOXA1 in bronchial epithelial cell proliferation.

HOXA1 has been shown to promote cell proliferation in adult hematopoietic stem cells and in various cancer cells. Although HOXA1 is expressed abundantly in the bronchial epithelium of human adult lungs, the function of HOXA1 in normal bronchial epithelial cells is not known [5,25]. To assess the function of HOXA1 in bronchial epithelial cells, we generated a HOXA1 K/O bronchial epithelial cell line using the CRISPR/Cas9 method followed by clonal expansion. This HOXA1 K/O cell line not only showed a truncated HOXA1 gene but also lacked HOXA1 expression at the protein level. The HOXA1 K/O bronchial epithelial cells generated smaller spheroids than the WT cells and also showed a delay in wound closure. These observations indicate that HOXA1 contributes to the proliferation of bronchial epithelial cells as observed in various cancer cell lines.

In carcinoma cell lines, HOXA1 regulates cell proliferation by various mechanisms depending on the cell line used. For example, in non-small cell lung cancer, HOXA1 promotes cell proliferation by activating the TGF-β/SMAD3 pathway [28]. In retinoblastoma cells, HOXA1 promotes cell proliferation via the Wnt/β-catenin signaling axis [29]. In gastric cancer cell lines, HOXA1 increases cell cycle progression by increasing cyclin D expression [27]. However, much less is known about the mechanisms of HOXA1-regulated proliferation in normal cells. To identify the possible mechanisms involved in HOXA1-reglated cell proliferation, we conducted a single-cell RNA-seq analysis, and the results indicated that the genes involved in cell cycle progression were downregulated in cells expressing low levels of HOXA1. The majority of the downregulated genes were related to cell cycle progression in HOXA1 K/O cells. Moreover, similar transcriptional changes in cell cycle progression were also observed in HOXA1 knockdown lung fibroblasts [30]. These results indicate that HOXA1 may promote cell proliferation by regulating cell cycle progression in normal cells. Consistent with these findings, HOXA1 K/O cells showed partial cell cycle arrest at the G0/G1 phase. Since HOXA1 K/O cells showed cells in the S and G2 phase, the DNA replication that occurs in the S phase and the protein expression that occurs in the G2 phase may be intact in these cells.

We observed a reduced expression of Cyclin E1 and Cyclin A2 in HOXA1 K/O cells. Cyclins are regulatory subunits of cyclin-dependent kinases (CDKs) and are sequentially synthesized and degraded during cell cycle progression. The activation of CDK2 by Cyclin E1 promotes the transitioning of cells from the G0/G1 to S phase, while CDK2 activated by Cyclin A2 is involved in the transition of cells from the S to G2 phase. Both phases are tightly regulated by the CDK inhibitory proteins p21/CDKN1A and p27/CDKN1B [31]. In the present study, we found that p21/CDKN1A, but not p27/CDKN1B, was significantly upregulated in HOXA1 K/O cells, indicting the role of p21/CDKN1A in cell cycle arrest at the G0/G1 and S phase in these cells. p21/CDKN1A expression is positively regulated by p53, a tumor suppressor protein. However, our ongoing studies indicated no difference in the total protein expression of p53. In cancer cells, HOXA1 overexpression has been shown to repress the expression of p21/CDKN1A independent of p53 [32]; therefore, it is possible that the HOXA1 regulation of p21/CDKN1A may not depend on p53 expression in bronchial epithelial cells.

Interestingly, the downregulated genes associated with cell cycle progression in HOXA1 K/O cells were also found to be downregulated in COPD compared to normal airway basal cells as determined by the analysis of the microarray database (GSE137557) [5]. Since COPD basal cells showed a two- to three-logs lower expression of HOXA1 mRNA than normal basal cells, it is possible that HOXA1 may affect the cell proliferation in COPD basal cells, which will be investigated in the near future.

In conclusion, our findings indicate that HOXA1 contributes to airway epithelial cell proliferation. Further, HOXA1 may regulate cell proliferation by promoting cell cycle progression from the Go/G1 phase to S phase, probably via the negative regulation of p21/CDKN1A.

## 4. Materials and Methods

### 4.1. Airway Epithelial Cell Line

The 16HBE14o cells were derived from normal bronchial epithelial cells and kindly provided by Prof. D Gruenert, University of California, San Francisco. The cells were cultured as described previously [33]. Briefly, cells were maintained in Modified Eagle Medium supplemented with 10% heat-inactivated fetal bovine serum, 10 mM L-glutamine, and 1% penicillin-streptomycin. The cells were routinely cultured in collagen-coated cell culture dishes to maintain the epithelial phenotype.

### 4.2. Generation of HOXA1 K/O Cells by CRISPR/Cas9 Method

Two single-guide RNAs (sgRNAs) targeting specific genomic loci were designed by using Benchling [Biology Software, https://benchling.com, accessed on 11 October 2021] design tools, and the sgRNAs with the least off-target effects were selected. The sgRNAs were synthesized by Synthego, Redwood City, CA, USA. The 16HBE14o- cells were grown to 70–80% confluency in a 10 cm culture dish, harvested using Trypsin/EDTA, and then suspended in electroporation buffer (Thermo Fisher Scientific, Waltham, MA, USA) at a density of 1.2 × 10^7^ cells per/mL. In the meantime, equal volumes of sgRNA (360 pmol) and Cas9 protein (180 pmol) (Aldevron, Fargo, ND, USA) were mixed and incubated at room temperature for 10 min to form the sgRNA/Cas9 complex. The cells were mixed with the sgRNA/Cas9 complex and electroporated using a Neon electroporation system (Thermo Fisher Scientific, Waltham, MA, USA) under the following conditions: 1400 V, 30 ms, 1 pulse. Cells were plated immediately following electroporation in complete medium for 48 to 72 h. Cells were clonally expanded by plating one cell per well in a 96-well plate, then passaged in duplicate upon confluence. PCR was performed to identify the HOXA1 K/O clone.

### 4.3. Genomic DNA Extraction and PCR Amplification of Targeted Loci

To assess the efficiency of the genome editing, genomic DNA was extracted from the treated cells using a Platinum Direct Universal master mix (Thermo Fisher Scientific) following the manufacturer’s instructions. PCR was performed to amplify the targeted genomic loci using primers designed to flank the Cas9 cleavage sites.

Forward Primer: CCTCGGACCATAGGATTACAAC

Reverse Primer: ACAGAACACTACCGTCACAATAA

The PCR reaction was carried out under the following conditions: 94 °C, 2 m; [94 °C, 15 s; 54 °C, 15 s; 68 °C, 20 s] × 35 cycles; 68 °C, 30 s. The PCR products were analyzed by agarose gel electrophoresis.

### 4.4. Analysis by Sanger Sequencing

To analyze deletions at the targeted loci, PCR products were purified and sequenced using Sanger sequencing (Genewiz, Burlington, MA, USA).

### 4.5. Spheroid Assay to Assess Cell Proliferation

An equal number of 16HBE14o- cells (1000 cells/well) were suspended in a 50% Corning^®^ Matrigel^®^ Growth Factor Reduced basement membrane matrix (Millipore Sigma, St. Louis, MO, USA) diluted in MEM complete medium, and plated in 6 mm trans wells. The final concentration of Matrigel^®^ was 25%. The plates were incubated for 5 days. The spheroids were imaged using phase contrast microscopy, and the number and size of the spheres were quantified by using ImageJ software version 1.54.

### 4.6. Kinetics of Wound Closure by Time-Lapse Microscopy

WT and HOXA1 K/O cells were grown to 100% confluence in 24-well plates. Wounds (5 mm × 2 mm) were created by a 1 mL pipette tip and imaged with a phase contrast microscope at 0, 6, 12, 18, and 24 h. The width of the wound was measured by ImageJ in the same area of the wound, and the results are presented as the percentage of wound closure as previously described [34].

### 4.7. Quantitation of Apoptotic and Necrotic Cells

The apoptosis and necrotic cells were quantified by using an apoptosis assay kit following the manufacturer’s instructions (BD Biosciences, San Jose, CA, USA). Briefly, WT and HOXA1 K/O cells were seeded at a low density (5 × 10^5^ cells/6 cm dish) and grown until the cells became 50% confluent. The cells were harvested along with floating cells and incubated with an antibody to Annexin V, a marker for apoptotic cells, and propidium iodide (a marker of necrotic cells and cells with a compromised plasma membrane). The cells were fixed and analyzed in a Symphony flow cytometer. The data were analyzed by FlowJo version 10 (Tree Star, Ashland, OR, USA).

### 4.8. Western Blot Analysis

Briefly, an equal amount of total protein from the cells was subjected to Western blot analysis with antibodies to 1:1000 diluted HOXA1 (LifeSpan Biosciences, Lynnwood, WA, USA), Cyclin A2, cyclin E1, p21, and p27 (Cell signaling Technology, Danvers, MA, USA). The blots were stripped and reprobed with GAPDH (Millipore Sigma, St. Louis, MO, USA) antibodies. The density of the protein bands was determined by ImageJ and expressed as fold change over GAPDH.

### 4.9. Cell Cycle Assay

The cell cycle progression of WT and HOXA1 K/O cells was assessed by flow cytometry as described previously [35]. Briefly, 5 × 10^5^ WT or HOXA1 K/O cells were plated in a 6 cm dish and cultured in complete medium for 24 h, and the medium was replaced and continued to culture for another 24 h. The medium was collected, and the cells were washed with PBS two times, and then the washes were mixed with the collected medium, centrifuged to collect all the floating cells. The cells in the dishes were collected by trypsinization and combined with floating cells. The cells were washed once with PBS and the cell pellet was finally suspended in 0.5 mL PBS and fixed by adding 4.5 mL cold 100% methanol and incubated at −20 °C for 20 min. Fixed cells were collected by centrifugation and incubated with 1 mL of 0.1 mg/mL RNAse A for 30 min on ice. Cells were recovered by centrifugation and incubated with 1 mL PBS containing 0.1% Triton X-100 and 50 µg/mL propidium iodide (Millipore Sigma) for 10 min on ice. The cells were washed and stored at 4 °C for up to 16 h prior to analysis. The cells were then analyzed in a Symphony Flow cytometer (BD Biosciences, San Jose, CA, USA). The data were analyzed by FlowJo version 10 (Tree Star, Ashland, OR, USA).

### 4.10. scRNA Sequencing

WT and HOXA1 K/O cells cultured in complete medium, until the cells reached 70 to 80% confluence. The cells were collected by trypsinization, centrifuged and suspended in complete culture medium, and mixed one–one and shipped on ice to GeneWiz/Azenta (South Plainfield, NJ, USA) for scRNA-sequencing according to their instructions for scRNA sequencing. The viability of the cells was >95% prior to loading on to the 10X Genomics Chromium controller, targeting 3000 cells per sample. The resulting libraries were sequenced on an Illumina HiSeq^®^ 4000 (San Diego, CA, USA) using 26 cycles for the first read and 98 cycles for the second read. Raw sequence data (bcl files) generated from Illumina were converted into fastq files and de-multiplexed using the 10X Genomics’ Cellranger mkfastq command. Subsequent UMI and cell barcode de-convolution was also performed prior to downstream analysis. Mapping to the respective genome and gene expression analysis was performed on Rosalind’s platform using Sauret for clustering cells based on HOXA1 expression and ClusterDE to identify differentially expressed genes between low and high HOXA1 expressing cells.

### 4.11. Statistical Analysis

All the cell culture experiments were performed at least in duplicate and repeated two to three times. Data are presented as median with range or average with SEM. If the data were normally distributed, statistical significance was assessed by a *t* Test or a one-way ANOVA to compare two groups or more than two groups, respectively. If the data were not normally distributed, a non-parametric analysis, the Mann–Whitney test to compare two groups, and ANOVA on ranks with the Kruskal–Wallace non-parametric test to compare three groups were used. Statistical analysis of the data was conducted using SigmaStat V4 (Grafiti, ST Palo Alto, CA, USA). A *p*-value of ≤0.05 was considered as statistically significant.

## Data Availability

All the data are provided in the manuscript. The original blots are provided in the Supplemental File. The RNAseq data have been submitted to Gene Expression Omnibus and the accession is pending.

## Supplementary Materials

The following supporting information can be downloaded at: https://www.mdpi.com/article/10.3390/ijms26052332/s1.

## Abbreviations

HOXA1	Homeobox A1
COPD	Chronic obstructive pulmonary disease
HBE	Human bronchial epithelial cells
CDKN1A	Cyclin dependent kinase 1A

## References

[1] Rock J.R., Onaitis M.W., Rawlins E.L., Lu Y., Clark C.P., Xue Y., Randell S.H., Hogan B.L. (2009). Basal cells as stem cells of the mouse trachea and human airway epithelium. Proc. Natl. Acad. Sci. USA.

[2] Rock J.R., Randell S.H., Hogan B.L. (2010). Airway basal stem cells: A perspective on their roles in epithelial homeostasis and remodeling. Dis. Model. Mech..

[3] Schneider D., Ganesan S., Comstock A.T., Meldrum C.A., Mahidhara R., Goldsmith A.M., Curtis J.L., Martinez F.J., Hershenson M.B., Sajjan U. (2010). Increased cytokine response of rhinovirus-infected airway epithelial cells in chronic obstructive pulmonary disease. Am. J. Respir. Crit. Care Med..

[4] Jing Y., Gimenes J.A., Mishra R., Pham D., Comstock A.T., Yu D., Sajjan U. (2019). NOTCH3 contributes to rhinovirus-induced goblet cell hyperplasia in COPD airway epithelial cells. Thorax.

[5] Pineau F., Shumyatsky G., Owuor N., Nalamala N., Kotnala S., Bolla S., Marchetti N., Kelsen S., Criner G.J., Sajjan U.S. (2020). Microarray analysis identifies defects in regenerative and immune response pathways in COPD airway basal cells. ERJ Open Res..

[6] Gohy S.T., Hupin C., Fregimilicka C., Detry B.R., Bouzin C., Gaide Chevronay H., Lecocq M., Weynand B., Ladjemi M.Z., Pierreux C.E. (2015). Imprinting of the COPD airway epithelium for dedifferentiation and mesenchymal transition. Eur. Respir. J..

[7] Ghosh M., Miller Y.E., Nakachi I., Kwon J.B., Baron A.E., Brantley A.E., Merrick D.T., Franklin W.A., Keith R.L., Vandivier R.W. (2018). Exhaustion of Airway Basal Progenitor Cells in Early and Established Chronic Obstructive Pulmonary Disease. Am. J. Respir. Crit. Care Med..

[8] Fossum S.L., Mutolo M.J., Yang R., Dang H., O’Neal W.K., Knowles M.R., Leir S.H., Harris A. (2014). Ets homologous factor regulates pathways controlling response to injury in airway epithelial cells. Nucleic Acids Res..

[9] Rock J.R., Gao X., Xue Y., Randell S.H., Kong Y.Y., Hogan B.L. (2011). Notch-dependent differentiation of adult airway basal stem cells. Cell Stem Cell.

[10] Gomi K., Arbelaez V., Crystal R.G., Walters M.S. (2015). Activation of NOTCH1 or NOTCH3 signaling skews human airway basal cell differentiation toward a secretory pathway. PLoS ONE.

[11] Gomi K., Staudt M.R., Salit J., Kaner R.J., Heldrich J., Rogalski A.M., Arbelaez V., Crystal R.G., Walters M.S. (2016). JAG1-Mediated Notch Signaling Regulates Secretory Cell Differentiation of the Human Airway Epithelium. Stem Cell Rev..

[12] Wang R., Ahmed J., Wang G., Hassan I., Strulovici-Barel Y., Hackett N.R., Crystal R.G. (2011). Down-regulation of the canonical Wnt beta-catenin pathway in the airway epithelium of healthy smokers and smokers with COPD. PLoS ONE.

[13] Danahay H., Pessotti A.D., Coote J., Montgomery B.E., Xia D., Wilson A., Yang H., Wang Z., Bevan L., Thomas C. (2015). Notch2 is required for inflammatory cytokine-driven goblet cell metaplasia in the lung. Cell Rep..

[14] Gao X., Bali A.S., Randell S.H., Hogan B.L. (2015). GRHL2 coordinates regeneration of a polarized mucociliary epithelium from basal stem cells. J. Cell Biol..

[15] Zhao R., Fallon T.R., Saladi S.V., Pardo-Saganta A., Villoria J., Mou H., Vinarsky V., Gonzalez-Celeiro M., Nunna N., Hariri L.P. (2014). Yap tunes airway epithelial size and architecture by regulating the identity, maintenance, and self-renewal of stem cells. Dev. Cell.

[16] McCluskey E.S., Liu N., Pandey A., Marchetti N., Kelsen S.G., Sajjan U.S. (2024). Quercetin improves epithelial regeneration from airway basal cells of COPD patients. Respir. Res..

[17] Wang K.C., Helms J.A., Chang H.Y. (2009). Regeneration, repair and remembering identity: The three Rs of Hox gene expression. Trends Cell Biol..

[18] Morgan R. (2006). Hox genes: A continuation of embryonic patterning?. Trends Genet..

[19] Leucht P., Kim J.B., Amasha R., James A.W., Girod S., Helms J.A. (2008). Embryonic origin and Hox status determine progenitor cell fate during adult bone regeneration. Development.

[20] Argiropoulos B., Humphries R.K. (2007). Hox genes in hematopoiesis and leukemogenesis. Oncogene.

[21] Takahashi Y., Hamada J., Murakawa K., Takada M., Tada M., Nogami I., Hayashi N., Nakamori S., Monden M., Miyamoto M. (2004). Expression profiles of 39 HOX genes in normal human adult organs and anaplastic thyroid cancer cell lines by quantitative real-time RT-PCR system. Exp. Cell Res..

[22] Yamamoto M., Takai D., Yamamoto F., Yamamoto F. (2003). Comprehensive expression profiling of highly homologous 39 hox genes in 26 different human adult tissues by the modified systematic multiplex RT-pCR method reveals tissue-specific expression pattern that suggests an important role of chromosomal structure in the regulation of hox gene expression in adult tissues. Gene Expr..

[23] Lebert-Ghali C.E., Fournier M., Kettyle L., Thompson A., Sauvageau G., Bijl J.J. (2016). Hoxa cluster genes determine the proliferative activity of adult mouse hematopoietic stem and progenitor cells. Blood.

[24] Xiong Z., Xia P., Zhu X., Geng J., Wang S., Ye B., Qin X., Qu Y., He L., Fan D. (2020). Glutamylation of deubiquitinase BAP1 controls self-renewal of hematopoietic stem cells and hematopoiesis. J. Exp. Med..

[25] Golpon H.A., Geraci M.W., Moore M.D., Miller H.L., Miller G.J., Tuder R.M., Voelkel N.F. (2001). HOX genes in human lung: Altered expression in primary pulmonary hypertension and emphysema. Am. J. Pathol..

[26] Li H., Wang X., Zhang M., Wang M., Zhang J., Ma S. (2020). Identification of HOXA1 as a Novel Biomarker in Prognosis of Head and Neck Squamous Cell Carcinoma. Front. Mol. Biosci..

[27] Yuan C., Zhu X., Han Y., Song C., Liu C., Lu S., Zhang M., Yu F., Peng Z., Zhou C. (2016). Elevated HOXA1 expression correlates with accelerated tumor cell proliferation and poor prognosis in gastric cancer partly via cyclin D1. J. Exp. Clin. Cancer Res..

[28] Qian Z., Zhang Q., Hu Y., Zhang T., Li J., Liu Z., Zheng H., Gao Y., Jia W., Hu A. (2018). Investigating the mechanism by which SMAD3 induces PAX6 transcription to promote the development of non-small cell lung cancer. Respir. Res..

[29] Lyv X., Wu F., Zhang H., Lu J., Wang L., Ma Y. (2020). Long Noncoding RNA ZFPM2-AS1 Knockdown Restrains the Development of Retinoblastoma by Modulating the MicroRNA-515/HOXA1/Wnt/beta-Catenin Axis. Investig. Ophthalmol. Vis. Sci..

[30] Trapnell C., Hendrickson D.G., Sauvageau M., Goff L., Rinn J.L., Pachter L. (2013). Differential analysis of gene regulation at transcript resolution with RNA-seq. Nat. Biotechnol..

[31] Fagundes R., Teixeira L.K. (2021). Cyclin E/CDK2: DNA Replication, Replication Stress and Genomic Instability. Front. Cell Dev. Biol..

[32] Zhang X., Zhu T., Chen Y., Mertani H.C., Lee K.O., Lobie P.E. (2003). Human growth hormone-regulated HOXA1 is a human mammary epithelial oncogene. J. Biol. Chem..

[33] Sajjan U., Wang Q., Zhao Y., Gruenert D.C., Hershenson M.B. (2008). Rhinovirus disrupts the barrier function of polarized airway epithelial cells. Am. J. Respir. Crit. Care Med..

[34] Faris A.N., Ganesan S., Chattoraj A., Chattoraj S.S., Comstock A.T., Unger B.L., Hershenson M.B., Sajjan U.S. (2016). Rhinovirus Delays Cell Repolarization in a Model of Injured/Regenerating Human Airway Epithelium. Am. J. Respir. Cell Mol. Biol..

[35] Maadi H., Soheilifar M.H., Wang Z. (2022). Analysis of Cell Cycle by Flow Cytometry. Methods Mol. Biol..

