# Supplementary material for: HOXA1 Contributes to Bronchial Epithelial Cell Cycle Progression by Regulating p21/CDKN1A
Source: Int J Mol Sci. 2025 Mar 5;26(5):2332. doi: 10.3390/ijms26052332 (PMC11899960; doi:10.3390/ijms26052332)

SUPPLEMENTAL MATERIAL

HOXA1 Contributes to Bronchial Epithelial Cell Cycle Progression by Regulating p21/CDKN1A

Elizabeth McCluskey [1,†], Sathesh Kanna Velli [1,†], Rafal Kaminski [2], Tyler Markward [1],

Hannah Leming [1], Daohai Yu [3] and Umadevi Sajjan [1,2,4,*]

Supplemental Table S1: Differentially regulated genes in HOXA1 K/O cells

Name	Description	Fold Chang	Log Fold C	p-Value	p-Adj	Average Lo	Cluster	low hoxa1	high hoxa1	Gene ID
AC058822.1		1.34834	0.431181	0.02618	0.999115	8.7289		9.13471	8.17213	
ATP8	ATP synthase F0 subunit 8	1.34088	0.42318	0.027296	0.999115	14.7187		15.0334	14.3156	4509
BACH2	BTB and CNC homology 1, basic leucine zipper transcript	1.34497	0.427577	0.028225	0.999115	9.2374		9.61306	8.73483	60468
BRIP1	BRCA1 interacting protein C-terminal helicase 1	1.35497	0.438262	0.023399	0.999115	11.7939		12.1317	11.3532	83990
C15orf53	chromosome 15 open reading frame 53	1.35399	0.437212	0.021188	0.999115	8.0901		8.55629	7.41392	400359
C8orf4	chromosome 8 open reading frame 4	1.36009	0.443701	0.021042	0.999115	8.4785		8.91906	7.85355	56892
CCNE2	cyclin E2	1.36896	0.453077	0.019756	0.999115	10.9009		11.2629	10.4187	9134
CDCA7	cell division cycle associated 7	1.32445	0.405391	0.035818	0.999115	10.8647		11.179	10.4643	83879
CTGF	connective tissue growth factor	1.36954	0.453689	0.01972	0.999115	10.6198		10.9868	10.129	1490
CYP1B1	cytochrome P450, family 1, subfamily B, polypeptide 1	1.30896	0.388424	0.045532	0.999115	9.7951		10.1111	9.39424	1545
DPYD	dihydropyrimidine dehydrogenase	1.35027	0.433245	0.025518	0.999115	10.8306		11.1729	10.3831	1806
DTL	denticleless E3 ubiquitin protein ligase homolog (Drosop	1.35242	0.435546	0.024272	0.999115	11.6644		12.0004	11.2268	51514
ENOX1	ecto-NOX disulfide-thiol exchanger 1	1.34291	0.425362	0.027655	0.999115	8.4851		8.89934	7.91369	55068
FAM111B	family with sequence similarity 111, member B	1.35776	0.441228	0.022872	0.999115	11.1555		11.5019	10.7004	374393
FHOD3	formin homology 2 domain containing 3	1.3343	0.416081	0.031665	0.999115	8.5248		8.92395	7.98243	80206
GLIS3	GLIS family zinc finger 3	1.33173	0.413298	0.03164	0.999115	8.2402		8.65662	7.6667	169792
GREB1L	growth regulation by estrogen in breast cancer-like	1.33458	0.416384	0.031478	0.999115	10.7752		11.1013	10.3553	80000
HIST1H2AH	histone cluster 1, H2ah	1.30841	0.387815	0.045269	0.999115	10.1302		10.4385	9.74085	85235
HIST1H4D	histone cluster 1, H4d	1.35143	0.434489	0.02581	0.999115	9.4388		9.81472	8.93497	8360
HIST1H4E	histone cluster 1, H4e	1.32536	0.406382	0.030207	0.999115	7.6812		8.14358	7.01917	8367
ID4	inhibitor of DNA binding 4, dominant negative helix-loop	1.32074	0.401349	0.03938	0.999115	9.4577		9.79618	9.02017	3400
KANK4	KN motif and ankyrin repeat domains 4	1.30321	0.382073	0.045005	0.999115	7.8103		8.22109	7.2529	163782
KIRREL3	kin of IRRE like 3 (Drosophila)	1.32159	0.402272	0.038185	0.999115	10.1174		10.4402	9.70436	84623
MAML2	mastermind-like transcriptional coactivator 2	1.31914	0.399593	0.038109	0.999115	11.0573		11.364	10.669	84441
MCM6	minichromosome maintenance complex component 6	1.36176	0.445468	0.021605	0.999115	11.2814		11.6309	10.821	4175
MMS22L	MMS22-like, DNA repair protein	1.30849	0.387899	0.043414	0.999115	11.2202		11.5146	10.8516	253714
MSH6	mutS homolog 6	1.35802	0.44151	0.022604	0.999115	11.5018		11.8451	11.0518	2956
MTRNR2L8	MT-RNR2-like 8	1.33067	0.412158	0.03266	0.999115	8.3377		8.74491	7.78137	1E+08
OLR1	oxidized low density lipoprotein (lectin-like) receptor 1	1.3029	0.38173	0.048995	0.999115	9.9168		10.2234	9.5307	4973
OMD	osteomodulin	1.29762	0.375866	0.039137	0.999115	7.174		7.65432	6.48171	4958
PLCB4	phospholipase C, beta 4	1.30697	0.38623	0.043671	0.999115	11.6717		11.9611	11.3103	5332
POLA1	polymerase (DNA directed), alpha 1, catalytic subunit	1.31399	0.393958	0.039942	0.999115	11.689		11.9853	11.3167	5422
RIMS2	regulating synaptic membrane exocytosis 2	1.33968	0.421888	0.028449	0.999115	12.0943		12.4143	11.6833	9699
SLC8A1	solute carrier family 8 (sodium/calcium exchanger), mem	1.35281	0.435956	0.025076	0.999115	9.0718		9.46496	8.53732	6546
SLFNL1	schlafen-like 1	1.35071	0.433713	0.025298	0.999115	8.7454		9.15357	8.18409	200172
TBC1D4	TBC1 domain family, member 4	1.30628	0.385467	0.047794	0.999115	8.724		9.07443	8.26965	9882
TSHZ2	teashirt zinc finger homeobox 2	1.3128	0.392652	0.043921	0.999115	8.9101		9.25985	8.4558	128553
UNG	uracil DNA glycosylase	1.3521	0.435205	0.025129	0.999115	10.497		10.8465	10.0377	7374
VAV3	vav 3 guanine nucleotide exchange factor	1.3093	0.388797	0.044858	0.999115	8.3643		8.73937	7.86845	10451
WDR76	WD repeat domain 76	1.31104	0.390715	0.042193	0.999115	11.1011		11.3991	10.7268	79968
ZFPM2	zinc finger protein, FOG family member 2	1.28894	0.366181	0.046363	0.999115	7.2082		7.66604	6.56456	23414
ANLN	anillin actin binding protein	-1.31328	-0.39318	0.039901	0.999115	12.132		11.7643	12.4253	54443
B4GALT3	UDP-Gal:betaGlcNAc beta 1,4- galactosyltransferase, po	-1.31717	-0.39744	0.041316	0.999115	9.4482		9.01611	9.78354	8703
BORA	bora, aurora kinase A activator	-1.3445	-0.42707	0.027799	0.999115	8.7625		8.21711	9.16239	79866
BRD8	bromodomain containing 8	-1.30226	-0.38101	0.047903	0.999115	10.6781		10.3109	10.9719	10902
BUB1B	BUB1 mitotic checkpoint serine/threonine kinase B	-1.36117	-0.44485	0.022191	0.999115	10.5203		10.0431	10.8799	701
CASC5	cancer susceptibility candidate 5	-1.30792	-0.38728	0.044037	0.999115	11.0136		10.6429	11.3095	57082
CDC42EP1	CDC42 effector protein (Rho GTPase binding) 1	-1.31494	-0.395	0.038833	0.999115	12.4601		12.0918	12.7536	11135
CDK1	cyclin-dependent kinase 1	-1.32982	-0.41123	0.032488	0.999115	11.8634		11.4668	12.1747	983
CEP55	centrosomal protein 55kDa	-1.33587	-0.41779	0.030415	0.999115	11.3579		10.9454	11.6793	55165
CKAP2	cytoskeleton associated protein 2	-1.36843	-0.45252	0.01963	0.999115	11.5304		11.0608	11.8848	26586
CKAP5	cytoskeleton associated protein 5	-1.34238	-0.42479	0.028731	0.999115	10.4037		9.95983	10.7443	9793
CSNK2B	casein kinase 2, beta polypeptide	-1.32032	-0.40088	0.036184	0.999115	12.6079		12.2319	12.9062	1460
CTDNEP1	CTD nuclear envelope phosphatase 1	-1.32743	-0.40864	0.033897	0.999115	11.4024		11.0048	11.7147	23399
DBF4	DBF4 zinc finger	-1.31522	-0.3953	0.039969	0.999115	11.1397		10.7589	11.4418	10926
DBF4B	DBF4 zinc finger B	-1.32922	-0.41058	0.035072	0.999115	9.5638		9.11347	9.90956	80174
DEPDC1B	DEP domain containing 1B	-1.3528	-0.43595	0.025265	0.999115	9.8463		9.36065	10.2114	55789
FAM134A	family with sequence similarity 134, member A	-1.30613	-0.3853	0.046409	0.999115	10.2443		9.86134	10.5486	79137
FOXM1	forkhead box M1	-1.35682	-0.44023	0.023187	0.999115	11.1214		10.6671	11.4675	2305
FST	follistatin	-1.32425	-0.40518	0.037485	0.999115	8.8905		8.40716	9.25659	10468
FZR1	fizzy/cell division cycle 20 related 1	-1.32837	-0.40965	0.03528	0.999115	8.8233		8.32454	9.19822	51343
GNB2	guanine nucleotide binding protein (G protein), beta pol	-1.3008	-0.3794	0.04662	0.999115	12.1987		11.8506	12.4794	2783
HJURP	Holliday junction recognition protein	-1.37043	-0.45462	0.019604	0.999115	10.2834		9.77874	10.6583	55355
HYLS1	hydrolethalus syndrome 1	-1.29518	-0.37315	0.041014	0.999115	7.1885		6.50061	7.6667	219844
IFNL1	interferon, lambda 1	-1.32429	-0.40522	0.037376	0.999115	8.8246		8.33558	9.19408	282618
JADE2	jade family PHD finger 2	-1.30705	-0.38631	0.047272	0.999115	9.3964		8.98135	9.7217	23338
KIAA2013	KIAA2013	-1.30709	-0.38636	0.046133	0.999115	10.0899		9.70113	10.3981	90231
KIF18A	kinesin family member 18A	-1.37151	-0.45576	0.018844	0.999115	9.0226		8.43139	9.44482	81930
KIF18B	kinesin family member 18B	-1.34296	-0.42541	0.028959	0.999115	9.8127		9.34615	10.1672	146909

KIF20B	kinesin family member 20B	-1.3557	-0.43904	0.023285	0.999115	11.5715		11.1264	11.9119	9585
KIF22	kinesin family member 22	-1.34035	-0.42261	0.028423	0.999115	11.7257		11.3099	12.0488	3835
KLK10	kallikrein-related peptidase 10	-1.31379	-0.39374	0.040265	0.999115	11.4931		11.119	11.7907	5655
KXD1	KxDL motif containing 1	-1.33831	-0.42041	0.029669	0.999115	11.1095		10.6888	11.4358	79036
MIIP	migration and invasion inhibitory protein	-1.32666	-0.4078	0.035418	0.999115	10.373		9.95745	10.697	60672
MLST8	MTOR associated protein, LST8 homolog	-1.31672	-0.39695	0.039212	0.999115	11.1354		10.752	11.439	64223
MXD3	MAX dimerization protein 3	-1.36013	-0.44375	0.022814	0.999115	9.76		9.25404	10.1364	83463
NUF2	NUF2, NDC80 kinetochore complex component	-1.34204	-0.42443	0.028477	0.999115	10.8373		10.4048	11.1709	83540
NUSAP1	nucleolar and spindle associated protein 1	-1.35969	-0.44328	0.021622	0.999115	12.8132		12.3722	13.1508	51203
OTUB1	OTU deubiquitinase, ubiquitin aldehyde binding 1	-1.30127	-0.37992	0.046737	0.999115	11.8274		11.476	12.1104	55611
OXA1L	oxidase (cytochrome c) assembly 1-like	-1.30449	-0.38348	0.045534	0.999115	11.3145		10.9531	11.6042	5018
PREB	prolactin regulatory element binding	-1.31269	-0.39252	0.042726	0.999115	10.2099		9.81472	10.5217	10113
RABGGTA	Rab geranylgeranyltransferase, alpha subunit	-1.32806	-0.40932	0.035434	0.999115	9.7946		9.35704	10.1324	5875
RANGAP1	Ran GTPase activating protein 1	-1.30587	-0.38501	0.044134	0.999115	11.8073		11.4487	12.0949	5905
RHNO1	RAD9-HUS1-RAD1 interacting nuclear orphan 1	-1.35425	-0.43749	0.024385	0.999115	10.5361		10.0731	10.8876	83695
SGOL1	shugoshin-like 1 (S. pombe)	-1.33703	-0.41903	0.031102	0.999115	10.1539		9.71248	10.4934	151648
SLPI	secretory leukocyte peptidase inhibitor	-1.30544	-0.38453	0.043398	0.999115	13.049		12.6978	13.3314	6590
SOX15	SRY (sex determining region Y)-box 15	-1.30111	-0.37975	0.047217	0.999115	11.5185		11.1646	11.8033	6665
STARD10	StAR-related lipid transfer (START) domain containing 10	-1.31146	-0.39117	0.040922	0.999115	12.0297		11.6641	12.3217	10809
TMEM41A	transmembrane protein 41A	-1.3062	-0.38538	0.046236	0.999115	10.3147		9.93341	10.6179	90407
TTK	TTK protein kinase	-1.34421	-0.42676	0.028061	0.999115	10.3708		9.92246	10.7141	7272
ZMAT5	zinc finger, matrin-type 5	-1.30398	-0.38292	0.04842	0.999115	9.8766		9.4866	10.1857	55954
ZNF358	zinc finger protein 358	-1.36276	-0.44654	0.021952	0.999115	9.9225		9.41895	10.2973	140467
ZNF581	zinc finger protein 581	-1.30737	-0.38667	0.046083	0.999115	10.0268		9.63559	10.3364	51545

Supplemental Figure S1 : Strategy for knockdown of HOXA1 in 16HBE14o- cells. A. Two sgRNA spanning 216 bp in Exon 1 were complexed with Cas9 enzyme and transfected into 16HBE14o-. The control cells were transfected with Cas9 enzyme alone. The cells were cultured for 72 h, genomic DNA was isolated and subjected to PCR and the PCR products were subjected to agarose gel electrophoresis. B. and C. The PCR products from control and HOXA1 sgRNA transfected cells were subjected to Sanger sequencing. The sequence of truncated band indicated deletion of 216 bp. Asterisk, the sequence retained in the CRISPR-HOXA1.

Supplemental Figure S 2: Time lapse microscopy to determine the kinetics of wound closure in WT and HOXA1 K/O cells. WT (8×10^4 cells/well) and HOXA1 K/O cells (1.6×10^5 cells/well) cells were seeded in 24 well plates and cultured until the cells reached 100% confluency. Mechanical wounds of approximately 1 x 6 mm were created, cells were washed with PBS and medium was replaced with fresh complete medium. The cultures were imaged under phase contrast microscope at 0, 6, 12, 18 and 24 h. the diameter of the wound was measured and expressed as % of wound closure over time after injury. Results represents mean ± S.D. calculated from 3 independent experiments done in triplicate wells (*p ≤0.05; unpaired t test).

Supplemental Figure S3: Quantification of apoptotic and necrotic cells in WT and HOXA1 K/O cells. The WT and HOXA1 K/O cells were seeded at low density (5×10^5 cells/6 cm dish) and cultured until the cells reached 50% confluence. The cells along with the floating cells were collected and incubated with FITC-labeled antibody to annexin V and propidium iodide. The cells were then fixed and anaylzed by flow cytometry. A. Gating strategy used for identification and quantification of apoptotic (left upper quadrangle, annexin V =ve cells), late apoptotic (right upper quadrangle, annexin V +ve and propidium iodide +ve cells) and necrotic (lower right quadrangle, propidium iodide +ve) cells. B to D. Quantification of apoptotic, late apoptotic and necrotic cells respectively. Results represents mean ± S.D. calculated from 3 independent experiments done in triplicate wells (*p ≤0.05; unpaired t test).

Supplemental Figure S4. Gating strategy used for detection of cells in G0/G1, S and G2 phase. HOXA1 K/O cells show cell cycle arrest at G0/G1 Phase. Equal number of WT and HOXA1 K/O cells were seeded in 6 mm dishes and cultured for 48 h with one media change at 24 h post-seeding. The cells were harvested, fixed in cold methanol, treated with RNase A, stained with propidium iodide and analyzed flow cytometry. The data was analyzed by FlowJo V10 to select the single cells and then histograms were generated with cell counts on the Y-axis and intensity of PI staining on the X-axis. Representative histograms of WT and HOXA1 K/O cells showing cells at different phase of cell cycle.

Supplemental Figure S1

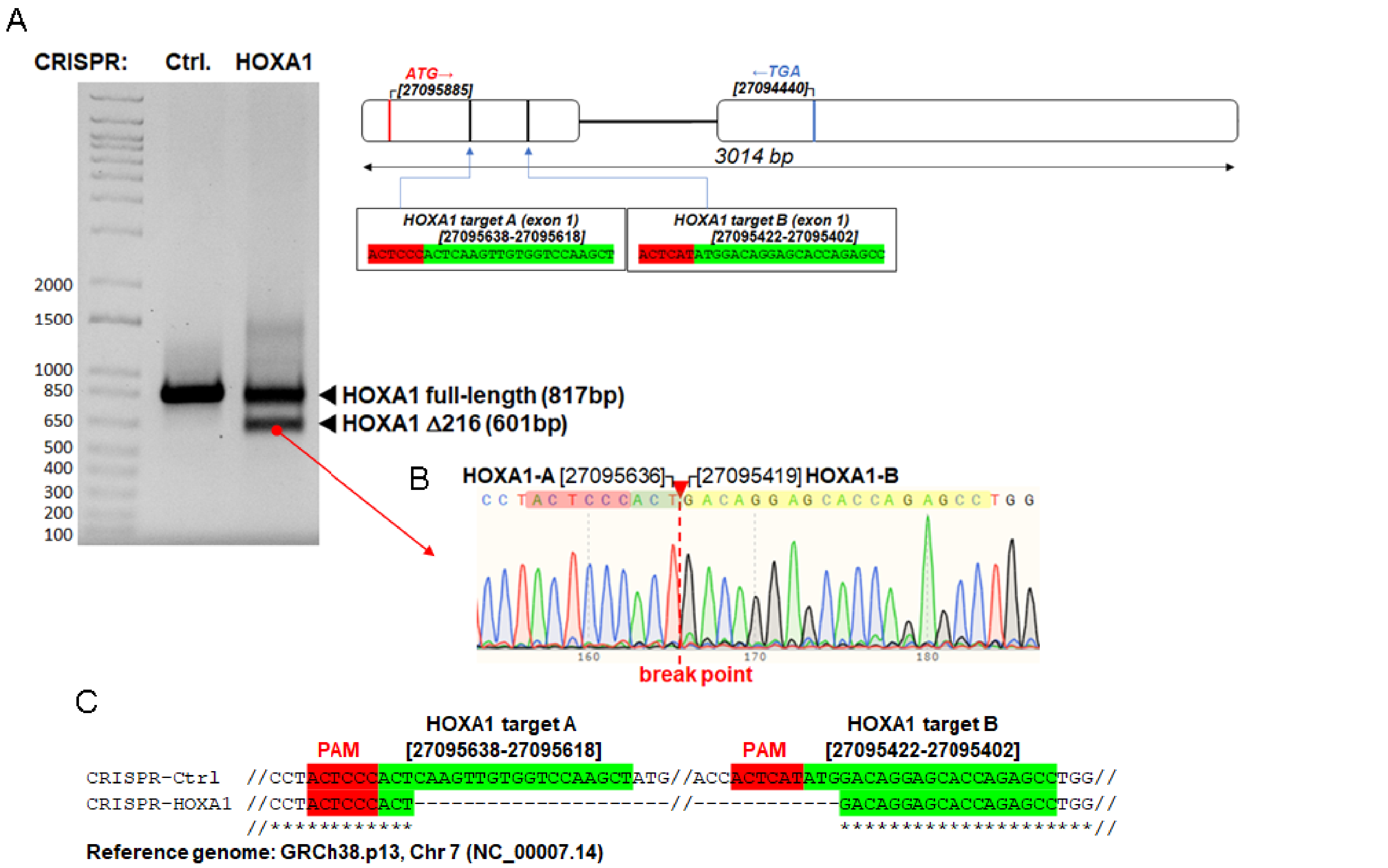

Supplemental Figure S2

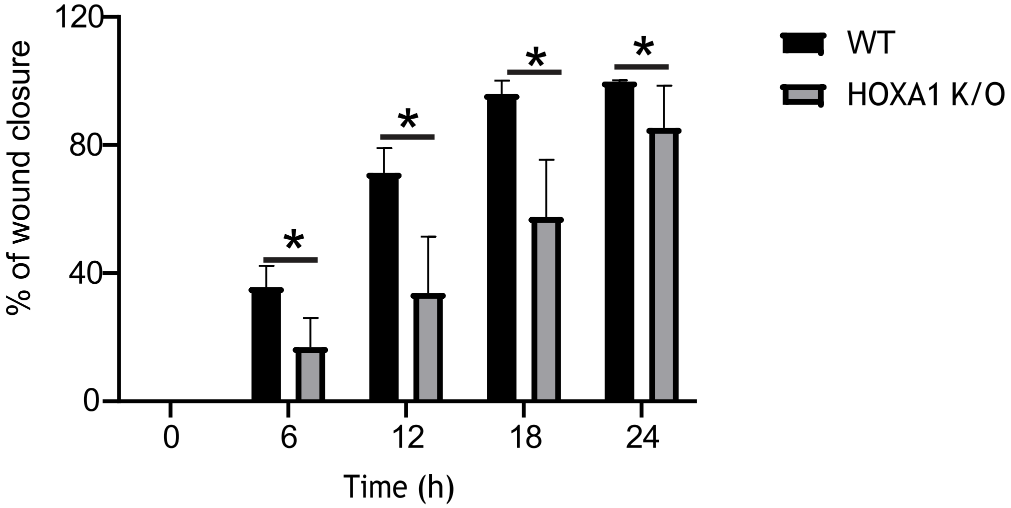

Supplemental Figure S3

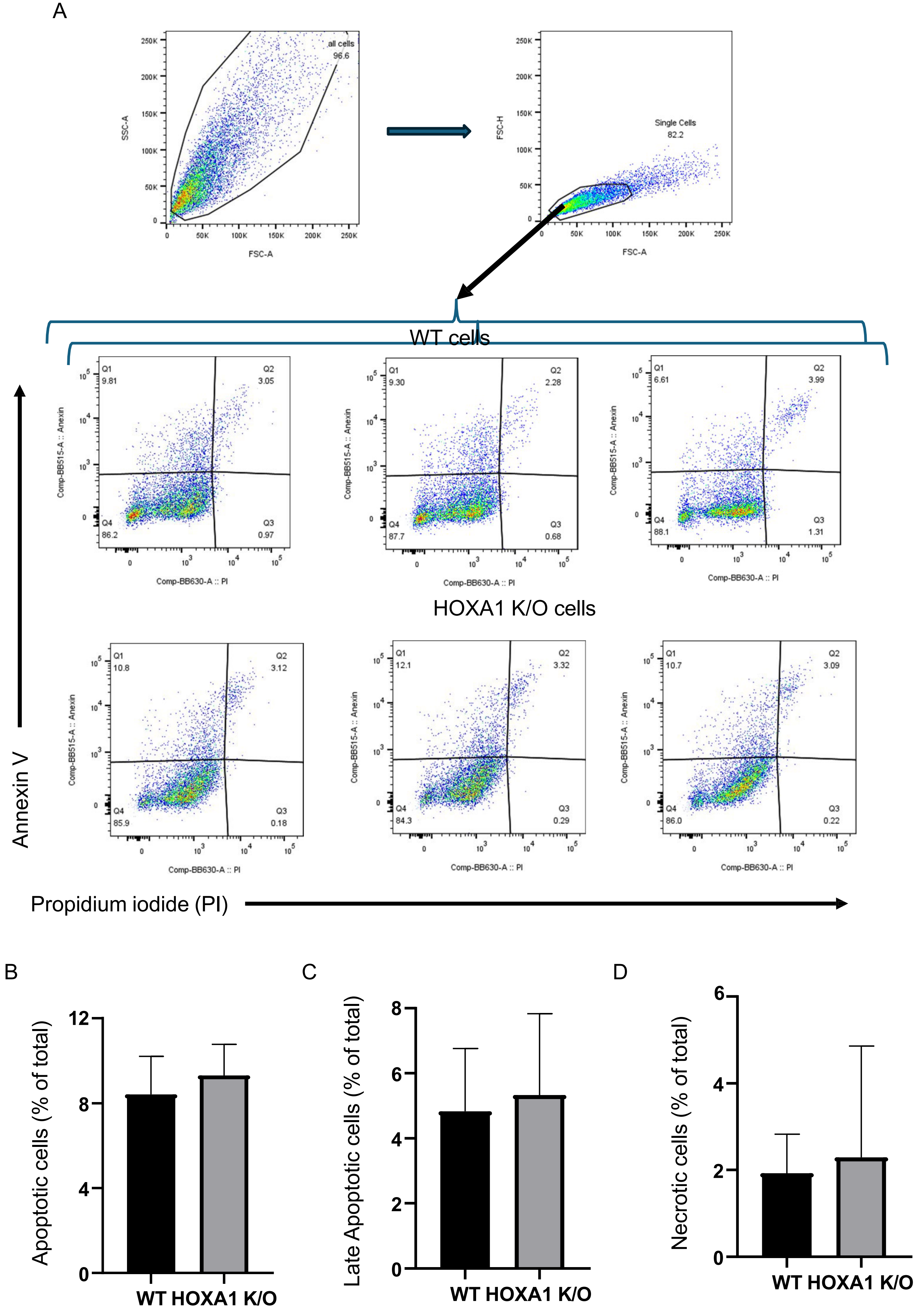

Supplemental Figure S4

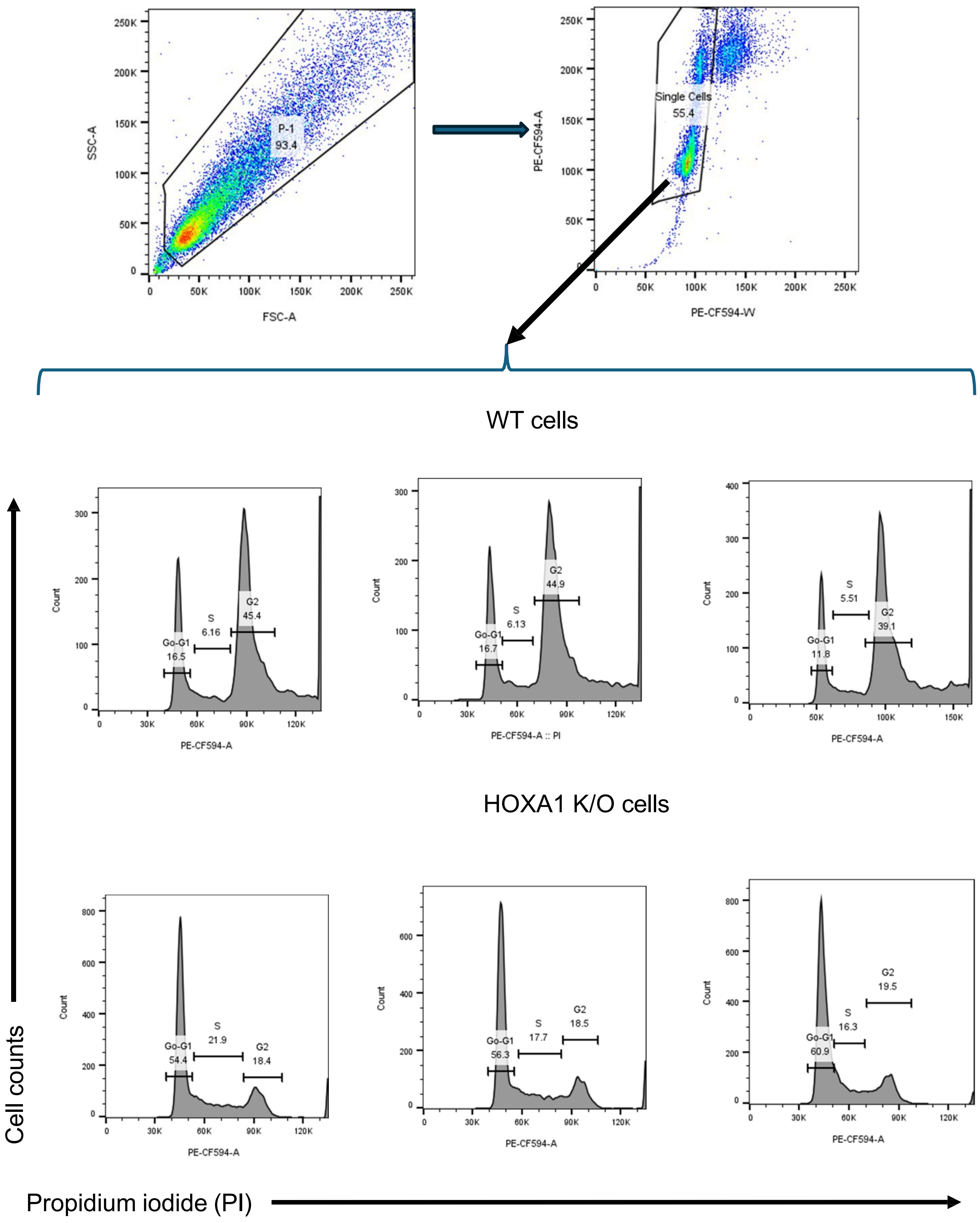

Supplement: Supplementary file 1 [file ijms-26-02332-s001.zip › ijms-3444863-supplementary.pdf]
